# Transcriptome and population structure of glassy-winged sharpshooters (*Homalodisca vitripennis*) with varying insecticide resistance in southern California

**DOI:** 10.1186/s12864-022-08939-1

**Published:** 2022-10-22

**Authors:** Cassandra L. Ettinger, Frank J. Byrne, Inaiara de Souza Pacheco, Dylan J. Brown, Linda L. Walling, Peter W. Atkinson, Richard A. Redak, Jason E. Stajich

**Affiliations:** 1 Department of Microbiology and Plant Pathology, University of California, Riverside, Riverside, CA USA; 2 Department of Entomology, University of California, Riverside, Riverside, CA USA; 3 Department of Botany and Plant Sciences, University of California, Riverside, Riverside, CA USA; 4 Institute for Integrative Genome Biology, University of California, Riverside, Riverside, CA USA

**Keywords:** Glassy-winged sharpshooter, Insecticide resistance, Transcriptome, RNA-seq, *Homalodisca vitripennis*, Neonicotinoids, Cytochrome P450s, Differentially expressed genes

## Abstract

**Background:**

*Homalodisca vitripennis* Germar*,* the glassy-winged sharpshooter, is an invasive insect in California and a critical threat to agriculture through its transmission of the plant pathogen, *Xylella fastidiosa*. Quarantine, broad-spectrum insecticides, and biological control have been used for population management of *H. vitripennis* since its invasion and subsequent proliferation throughout California. Recently wide-spread neonicotinoid resistance has been detected in populations of *H. vitripennis* in the southern portions of California’s Central Valley. In order to better understand potential mechanisms of *H. vitripennis* neonicotinoid resistance, we performed RNA sequencing on wild-caught insecticide-resistant and relatively susceptible sharpshooters to profile their transcriptome and population structure.

**Results:**

We identified 81 differentially expressed genes with higher expression in resistant individuals. The significant largest differentially expressed candidate gene linked to resistance status was a cytochrome P450 gene with similarity to CYP6A9. Furthermore, we observed an over-enrichment of GO terms representing functions supportive of roles in resistance mechanisms (cytochrome P450s, M13 peptidases, and cuticle structural proteins). Finally, we saw no evidence of broad-scale population structure, perhaps due to *H. vitripennis'* relatively recent introduction to California or due to the relatively small geographic scale investigated here.

**Conclusions:**

In this work, we characterized the transcriptome of insecticide-resistant and susceptible *H. vitripennis* and identified candidate genes that may be involved in resistance mechanisms for this species. Future work should seek to build on the transcriptome profiling performed here to confirm the role of the identified genes, particularly the cytochrome P450, in resistance in *H. vitripennis*. We hope this work helps aid future population management strategies for this and other species with growing insecticide resistance.

**Supplementary Information:**

The online version contains supplementary material available at 10.1186/s12864-022-08939-1.

## Background

The glassy-winged sharpshooter (GWSS), *Homalodisca vitripennis* Germar (Hemiptera: Cicadellidae), is a xylem-feeding leafhopper, which is invasive to California, and has proliferated since its introduction in the 1990s [1, 2]. GWSS has a broad host range, with over 340 reported plant species according to the California Department of Food and Agriculture (CDFA) (https://www.cdfa.ca.gov/pdcp/Documents/HostListCommon.pdf). In California’s agricultural systems, citrus is the major feeding, over-wintering, and reproductive host for GWSS, and the distribution of the insect is closely associated with the major citrus-growing regions in the state [2]. Although citrus can sustain high densities of GWSS, the major economic impact of the insect is as a vector of several strains of the xylem-limited, plant pathogenic bacterium, *Xylella fastidiosa*. This pathogen is the causal agent of several important diseases of crops in California, including Pierce's disease (PD) of grapes, oleander leaf scorch, and almond leaf scorch, and outside of California is also responsible for Citrus variegated chlorosis [3]. In particular, contiguous plantings of citrus and grapes have resulted in a significant increase in the incidence of PD, given the annual movement of GWSS from citrus to adjacent vineyards during the spring when grapes come out of dormancy [4].

The introduction of GWSS to California initially led to outbreaks of the destructive PD in the Temecula valley [1, 5, 6]. To combat the threat to the viticulture industry, area-wide treatments with the systemic neonicotinoid insecticide, imidacloprid, were undertaken and were successful at reducing the population sizes in both the southern Central Valley and Southern California in general [5, 6]. As GWSS began to spread and proliferate in the California Central Valley during the early 2000s, area-wide treatments with neonicotinoids (acetamiprid and imidacloprid) and pyrethroids were also introduced in Tulare and Kern counties [7]. The area-wide programs were highly successful until about 2012, when the levels of control appeared to be compromised [7, 8]. Recent work has found that applications of neonicotinoids have led to high levels of resistance in some GWSS populations, and it is believed that this was one of the major contributing factors to the population resurgence in the region [9, 10].

Insecticide resistance generally occurs through several co-existing processes spanning behavioral (e.g. avoidance), and physiological mechanisms (e.g. cuticle modifications, detoxification by host or symbionts, and target site alterations) [11–13]. Investigations of neonicotinoid resistance have consistently found enhanced detoxification by constitutively overexpressed cytochrome P450 monooxygenases in many insects spanning members of the orders Coleoptera (e.g., *Tribolium castaneum, Leptinotarsa decemlineata*), Diptera (e.g., *Bradysia odoriphaga*) and Hemiptera (e.g., *Bemisia tabaci*, *Myzus persicae*, *Laodelphax striatellus, Rhopalosiphum padi,* and *Nilaparvata lugens*) [14–23]. Transcriptome-based profiling approaches have enabled a broader understanding of resistance mechanisms, with many insects, in addition to having up-regulated cytochrome P450s, displaying differential expression of genes with functions related to cuticle structure and assembly, and detoxification through esterases, glutathione-S-transferases, or ABC transporters [24–31].

Despite the high levels of neonicotinoid and especially imidacloprid resistance observed in several populations of California GWSS, little is known about the underlying mechanisms involved in conferring resistance in these populations. In this study, we sought to profile the transcriptome of wild-caught resistant and susceptible GWSS obtained from different southern California populations. Specifically, we sought to (i) identify differentially expressed genes (DEGs) between resistant and susceptible populations, (ii) assess DEGs for functional enrichment that might relate to resistance mechanisms, and (iii) assess population structure in coding regions between resistant and susceptible populations to order to identify single nucleotide variants (SNVs) that may correlate with resistance status. Based on previous studies of neonicotinoid resistance, we hypothesized that we would see upregulation of one or more cytochrome P450s, as well as other genes related to cuticle modifications and detoxification. Understanding expression patterns of gene candidates linked to insecticide resistance in GWSS may help inform long-term solutions for population management of this species.

## Results

### Transcriptome identifies gene candidates linked to insecticide-resistance status

**Fig. 1:**
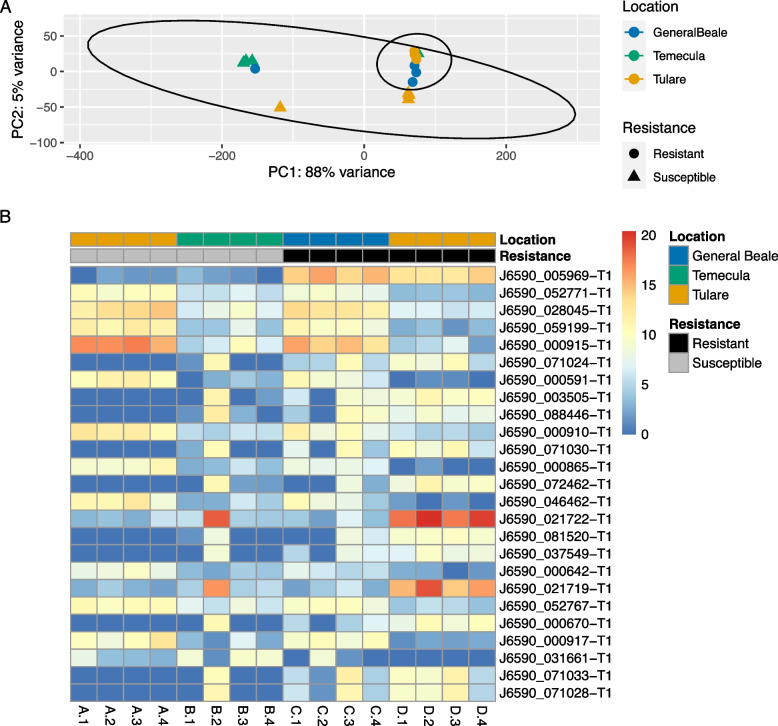
Gene expression differs between GWSS populations and with resistance status. **A** Principal Component Analysis (PCA) of variance stabilized transcriptomic count data representing the full dataset. Each point represents an individual transcriptome sample. Samples are colored by collection location, while shapes are used to display resistance status (circle = resistant, triangle = susceptible). Resistant and susceptible populations are further highlighted by ellipses representing the 95% confidence interval around the centroid of each group. **B** Heatmap showing the variance stabilized counts of the 25 most significant DEG between resistant and susceptible populations. Each column represents a GWSS RNAseq sample with letters (A-D) representing populations and numbers (1–4) representing replicate (see Table 1 for additional sample information)

Using DESeq2, we identified 607 DEGs between resistant and susceptible GWSS populations (Table S1). Of these, 81 had higher expression in resistant populations and 526 had higher expression in susceptible populations. Of the DEGs, 57.3% had a functional annotation match to at least one database. Insect cuticle proteins were the dominant function of DEGs with higher expression in susceptible populations (IPR000618; *n* = 28). In contrast, M13 peptidases (IPR000718; *n* = 11) and cytochrome P450s (IPR001128; *n* = 8) were the dominant functions found with higher expression in resistant populations. Ordination of overall transcriptome profiles did not depict a strong pattern related to resistance and while many genes were identified as differentially expressed, only a single gene had an obvious consistent pattern linked to resistance status, J6590_005969 (Fig. 1).

**Fig. 2:**
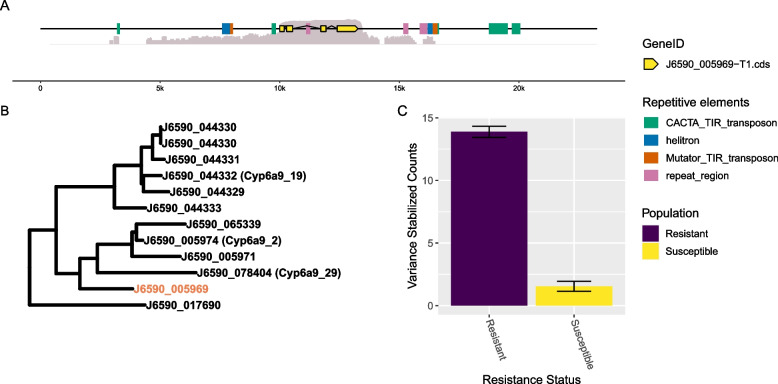
A single cytochrome P450 (J6590_005969) is most closely linked to resistance status. **A** Depiction of the architecture of the genomic region housing J6590_005969, including the 10 kb up and downstream of the candidate gene. Log-transformed coverage from resistant individuals is mapped to the region in gray. The gene (exons) is displayed in yellow. Repetitive elements as identified and annotated by EDTA are also shown in green (CACTA TIR transposon), blue (helitron), orange (Mutator TIR transposon) and pink (simple repetitive region). **B** Phylogeny of cytochrome P450s in the GWSS genome. J6590_005969 is highlighted in the tree in orange. A full phylogeny of cytochrome P450s in GWSS and their respective transcriptomic expression across samples can be found in Figure S1. **C** Mean of variance stabilized counts of reads mapping to J6590_005969 in resistant and susceptible populations. Bars represent standard error

Based on previous insecticide literature [14–23], we had hypothesized that cytochrome P450 genes might be partially or fully responsible for insecticide resistance of GWSS in the California Central Valley. Of the 81 DEGs identified as having higher expression in the resistant populations, eight were annotated as cytochrome P450s. Of these, a single gene, J6590_005969, was also the most significantly differentially expressed gene between resistant and susceptible populations (Fig. 2). Phylogenetic approaches place J6590_005969 in a clade with other GWSS cytochrome P450s belonging to the CYP6A9 family. Interestingly, this locus is flanked on both sides by Helitron, Mutator and CACTA transposons, similar to other observations of repetitive elements flanking cytochrome P450s involved in detoxification (e.g., [32]). None of the elements surrounding J6590_005969 were predicted by the Extensive de novo TE Annotator (EDTA) to be structurally intact (i.e. containing TIR regions, transposases or protein domains).

### Functional enrichment of GO terms supports a landscape of resistance in GWSS

**Fig. 3:**
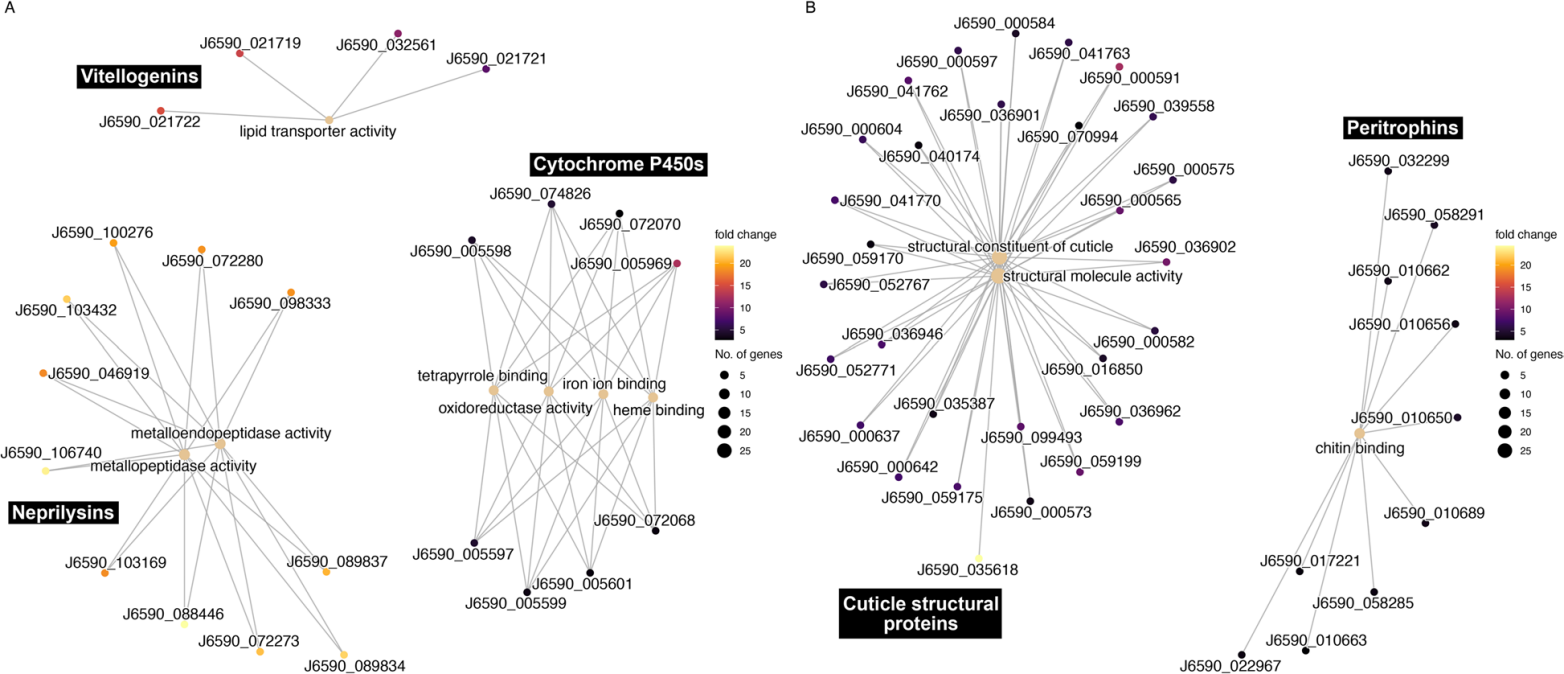
Gene concept network showing links between genes with shared over-enriched molecular function GO terms. **A** Over-enriched GO terms from the molecular function (MF) category representing genes with higher expression in resistant GWSS populations. **B** Over-enriched MF GO terms representing genes with higher expression in susceptible GWSS populations. Each cluster is annotated with the dominant gene function as determined by the gene annotation and/or from best matches to UniProt (black boxes)

GO term enrichment analysis was performed on the DEGs to identify significantly over-enriched functional terms (Table 2, Bonferroni-adjusted *p* < 0.05). For DEGs with higher expression in insecticide-resistant GWSS, we found that GO terms for seven molecular functions (MF) and 15 biological processes (BP) were over-enriched, but no terms for cellular compartments (CC) were enriched. For DEGs with higher expression in susceptible GWSS, we found that GO terms for three MFs and three CCs were over-enriched, but found no enrichment in terms for BPs. Generally, over-enriched MF terms in resistant GWSS were represented by gene clusters with predicted functions such as cytochrome P450s (GO:0005506, GO:0016705, GO:0020037, GO:0046906), the M13 protease neprilysin (GO:0004222, GO:0008237) and vitellogenins (GO:0005319) (Fig. 3). In contrast, gene clusters represented by over-enriched MF terms in susceptible GWSS were dominated by genes with functions related to cuticle structural proteins (GO:0042302, GO:0005198) and peritrophins (GO:0008061). The functions of genes represented in over-enriched BP terms in insecticide-resistant GWSS include vitellogenins (GO:0006869,GO:0010876) and a cluster of three gene copies of the body color gene *yellow* (GO:0018958, GO:0046189, GO:0042440, GO:0046148, GO:0019953, GO:0032504, GO:0044703, GO:0048609, GO:0051704, GO:0000003, GO:0022414, GO:1901617, GO:1901615). The functions of over-enriched CC terms in susceptible insects include peritrophins (GO:0005576), and NADH-ubiquinone oxidoreductases and MICOS complex subunits (GO:0019866, GO:0005743).

### No evidence of broad-scale population structure from coding region variants

**Fig. 4:**
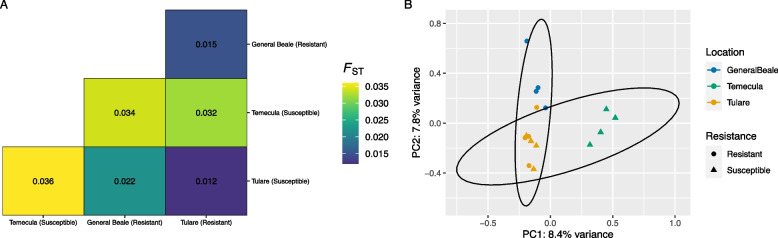
Low population differentiation across GWSS individuals from different collection locations and with different resistant statuses. **A** Pairwise F_ST_ values are shown here as heatmap with each value shown and F_ST_ values colored on a gradient with lowest values in blue and higher values in yellow. **B** PCA of population variation based on the SNV data. Each point represents an individual variant profile. Samples are colored by collection location while shapes are used to display resistance status (circle = resistant, triangle = susceptible). Resistant and susceptible populations are further highlighted by ellipses representing the 95% confidence interval around the centroid of each group

Observed heterozygosity was significantly lower than expected (Bartlett’s test, *p* < 0.001) and F_IS_ was 0.25 indicating high levels of non-random mating (e.g. inbreeding) in sampled GWSS. Overall F_st_ was 0.02, indicating low differentiation between populations (i.e., A-D; Table 1) and possibly high levels of migration or gene flow between populations, which is consistent with the relatively small geographic range studied here. Within-population F_ST_ were 0.028 and 0.033 for resistant and susceptible populations, respectively, and were 0.070, 0.081, 0.087, and 0.033 for population A (Tulare susceptible), B (Temecula susceptible), C (General Beale resistant) and D (Tulare resistant), respectively. Pairwise (between-population) F_ST_ was 0.011 between resistant and susceptible populations. Pairwise F_ST_ was similarly low for between A, B, C and D populations, ranging from 0.012 to 0.036 (Fig. 4).

**Table 1:** Sample information

Sample Code	Population Code	Replicate no	Original Sample Code	Imidacloprid Status	Collection Location	Agricultural Method	Host plant species	Collection Date	GPS
A1	A	1	A1	Susceptible	Tulare	Organic	*Citrus sinensis* (L.) Osbeck	2019–08-23	35.985913 N 118.968921 W
A2	A	2	A2	Susceptible	Tulare	Organic	*Citrus sinensis* (L.) Osbeck	2019–08-23	35.985913 N 118.968921 W
A3	A	3	A3	Susceptible	Tulare	Organic	*Citrus sinensis* (L.) Osbeck	2019–08-23	35.985913 N 118.968921 W
A4	A	4	A5	Susceptible	Tulare	Organic	*Citrus sinensis* (L.) Osbeck	2019–08-23	35.985913 N 118.968921 W
B1	B	1	B1	Susceptible	Temecula	Organic	*Citrus paradisi* Macfadyen	2019–08-20	33.5258405 N 117.0392916 W
B2	B	2	B2	Susceptible	Temecula	Organic	*Citrus paradisi* Macfadyen	2019–08-20	33.5258405 N 117.0392916 W
B3	B	3	B3	Susceptible	Temecula	Organic	*Citrus paradisi* Macfadyen	2019–08-20	33.5258405 N 117.0392916 W
B4	B	4	B5	Susceptible	Temecula	Organic	*Citrus paradisi* Macfadyen	2019–08-20	33.5258405 N 117.0392916 W
C1	C	1	C5	Resistant	General Beale	Organic	*Citrus limon* (L.) Burm	2019–08-30	35.2696228 N 118.7613945 W
C2	C	2	C6	Resistant	General Beale	Organic	*Citrus limon* (L.) Burm	2019–08-30	35.2696228 N 118.7613945 W
C3	C	3	C7	Resistant	General Beale	Organic	*Citrus limon* (L.) Burm	2019–08-30	35.2696228 N 118.7613945 W
C4	C	4	C8	Resistant	General Beale	Organic	*Citrus limon* (L.) Burm	2019–08-30	35.2696228 N 118.7613945 W
D1	D	1	D5	Resistant	Tulare	Conventional	*Citrus limon* (L.) Burm	2019–08-16	35.9641278 N 118.9620478 W
D2	D	2	D6	Resistant	Tulare	Conventional	*Citrus limon* (L.) Burm	2019–08-16	35.9641278 N 118.9620478 W
D3	D	3	D7	Resistant	Tulare	Conventional	*Citrus limon* (L.) Burm	2019–08-16	35.9641278 N 118.9620478 W
D4	D	4	D8	Resistant	Tulare	Conventional	*Citrus limon* (L.) Burm	2019–08-16	35.9641278 N 118.9620478 W

Specifics of each individual GWSS, including the sample code referred to in this study, population code, replicate number, original sample code upon collection, imidacloprid status, general collection location, agricultural method at each collection site, host plant species, collection date and GPS coordinates of each collection site

Further, population structure results from fastSTRUCTURE (marginal likelihood maximized at K = 1, Figure S2) and Landscape and Ecological Association Studies (LEA) (lowest cross-entropy at K = 1; Tracy Widom test *p* > 0.05 for all PCA eigenvalues, Figure S3) were both supportive of weak differentiation between populations. Despite a lack of overall population structure, using PCA ordinations, we observed some minimal separation by the general location individuals were collected from (Fig. 4). Thus, when performing analysis of molecular variance (AMOVA) tests, we used resistance status alone (therefore assuming no population structure) and in a nested structure to account for separation by collection location or population. AMOVA tests on resistance status alone were significant (*p* < 0.01), but only explained a tiny portion, 1.78%, of observed variation. Most of the variation, 98.22%, was found within individuals, further supporting a panmictic (randomly mating) population. When using a nested structure, AMOVA tests of resistance status were not significant (*p* > 0.05), indicating no detectable signal of resistance when accounting for local population structure. In these cases, collection location or population were significant (*p* < 0.01), accounting for 4.05% of variation, indicating that while there is no detectable broad-scale population structure, there may still be some local adaptation occurring. We used OutFlank to identify F_st_ outliers as likely variants potentially under selection due to resistance status, but no significant outliers were detected (*q* > 0.1).

We used SNPEff annotations of the functional effect of variants to identify SNVs that might contribute to the increased expression of J6590_005969. Unfortunately, the low levels of expression of J6590_005969 in susceptible GWSS prevented our ability to call SNVs in this gene for susceptible samples. However, given that the reference genome represents a susceptible genotype [33], we still investigated the variants called in the resistant GWSS individuals. We found no SNVs that were homozygous or heterozygous for an alternate allele that was predicted to affect J6590_005969 function.

## Discussion

### Transcriptome results identified a cytochrome P450 candidate strongly linked to resistance

As has been seen in previous neonicotinoid resistance studies [14–23], we observed eight cytochrome P450s with higher expression in insecticide-resistant GWSS, with a robust pattern of expression in a single overexpressed cytochrome P450, J6590_005969, similar to CYP6A9, linked to resistance status. This gene candidate was flanked by repetitive elements, whose presence was previously hypothesized to be characteristic of xenobiotic-metabolizing P450s [32]. The mechanisms behind overexpression of many cytochrome P450s vary and part of our inability to identify SNVs responsible for J6590_005969’s higher expression may be because our analyses focused on coding regions. In other insects, overexpression of cytochrome P450s has been found to be regulated by *trans* and/or *cis* regulatory elements [34–36]. In *N. lugens*, for example, SNVs in the promoter region of CYP6AY1 were observed to enhance promoter activity and hypothesized to be acting as *cis*-acting factors that enhance expression in resistant individuals [37]. In *Drosophila melanogaster,* the characterization of several *CYP6A* genes with higher expression in resistant individuals suggested that a defective repressor might be involved in regulation of DDT resistance [38]. Future work should focus on confirming J6590_005969’s role in resistance and characterizing the upstream promoter region of this gene to investigate the molecular mechanism driving its overexpression in resistant individuals. New advances demonstrating the efficiency of using CRISPR mutagenesis in GWSS may dramatically accelerate opportunities to examine the functional capabilities of J6590_005969 in vivo [39, 40].

### Functional enrichment suggests a complex multi-gene response to insecticides

The transcriptome of GWSS has been previously described as being dominated by genes annotated with GO terms related to molecular binding, catalytic (e.g., hydrolase and oxidoreductases), and transporter activity [41]. These categories broadly match with the GO terms observed to be over-enriched in insecticide-resistant populations here (Table 2). Specifically, in addition to cytochrome P450s, we characterized an overall transcriptome pattern in resistant individuals supportive of additional genes being directly involved in or indirectly affected by insecticide resistance mechanisms including genes related to detoxification, immune response and cuticle modifications. Genes indirectly affected by resistance mechanisms may be the result of trade-offs and represent a potential fitness cost of insecticide resistance [42–46]. Although we were able to observe an over-enrichment of functions that may be related to resistance, 42.7% of DEGs had no functional annotation. Additional novel genes or functions may be involved in resistance that are not discussed here. Studies focused on the molecular characterization of unannotated genes in this and other insect species are needed to help close these annotation gaps.

**Table 2:** Over-enriched GO terms for genes that are differentially expressed between resistant and susceptible GWSS

Insecticide Status	GO Category	GO ID	GO Term	*p*-adjusted	*q*-value	Gene count
Resistant	MF	GO:0004222	metalloendopeptidase activity	< 0.001	< 0.001	11
Resistant	MF	GO:0008237	metallopeptidase activity	< 0.001	< 0.001	11
Resistant	MF	GO:0005506	iron ion binding	< 0.001	< 0.001	8
Resistant	MF	GO:0016705	oxidoreductase activity, acting on paired donors, with incorporation or reduction of molecular oxygen	< 0.001	< 0.001	8
Resistant	MF	GO:0020037	heme binding	< 0.001	< 0.001	8
Resistant	MF	GO:0046906	tetrapyrrole binding	< 0.001	< 0.001	8
Resistant	MF	GO:0005319	lipid transporter activity	< 0.001	< 0.001	4
Susceptible	MF	GO:0042302	structural constituent of cuticle	< 0.001	< 0.001	28
Susceptible	MF	GO:0005198	structural molecule activity	< 0.001	< 0.001	29
Susceptible	MF	GO:0008061	chitin binding	< 0.001	< 0.001	10
Resistant	BP	GO:0018958	phenol-containing compound metabolic process	< 0.001	< 0.001	3
Resistant	BP	GO:0046189	phenol-containing compound biosynthetic process	< 0.001	< 0.001	3
Resistant	BP	GO:0042440	pigment metabolic process	0.001	< 0.001	3
Resistant	BP	GO:0046148	pigment biosynthetic process	0.001	< 0.001	3
Resistant	BP	GO:0019953	sexual reproduction	0.001	< 0.001	3
Resistant	BP	GO:0032504	multicellular organism reproduction	0.001	< 0.001	3
Resistant	BP	GO:0044703	multi-organism reproductive process	0.001	< 0.001	3
Resistant	BP	GO:0048609	multicellular organismal reproductive process	0.001	< 0.001	3
Resistant	BP	GO:0051704	multi-organism process	0.001	< 0.001	3
Resistant	BP	GO:0006869	lipid transport	0.001	< 0.001	4
Resistant	BP	GO:0010876	lipid localization	0.001	< 0.001	4
Resistant	BP	GO:0000003	reproduction	0.003	< 0.001	3
Resistant	BP	GO:0022414	reproductive process	0.003	< 0.001	3
Resistant	BP	GO:1901617	organic hydroxy compound biosynthetic process	0.004	< 0.001	3
Resistant	BP	GO:1901615	organic hydroxy compound metabolic process	0.021	< 0.001	3
Susceptible	BP	none	none	none	none	none
Resistant	CC	none	none	none	none	none
Susceptible	CC	GO:0005576	extracellular region	< 0.001	< 0.001	13
Susceptible	CC	GO:0005743	mitochondrial inner membrane	0.032	0.011	4
Susceptible	CC	GO:0019866	organelle inner membrane	0.044	0.011	4

Enrichment analysis of molecular functions of genes differentially expressed between resistant and susceptible GWSS identified GO terms that were significantly over-enriched (Bonferroni adjusted *p* < 0.05). Here we provide whether the term was enriched in genes with higher expression (based on log_2_ fold change) in resistant or susceptible GWSS populations, the GO category (MF: molecular function, BP: biological process, CC: cellular component), GO ID, GO term, the Bonferroni adjusted *p*-value, the *q*-value and the gene count for each enriched term cluster. GO categories with no over-enriched terms are represented in the table using ‘none’

We observed upregulation of neprilysin (M13 peptidases) and vitellogenin-like genes in resistant GWSS. Neprilysin and neprilysin-like proteins are zinc metalloendopeptidases and are type II integral membrane proteins that turn off signaling events at the cell surface [47]. Previous studies in insects have seen altered expression of M13 peptidases during metamorphosis and immune responses [48–51]. Vitellogenins are important for insect reproduction. They play a role in immune responses and protect against oxidative stress induced by insecticides in bees [52–54]. Additionally, the expression of vitellogenins in the white-backed planthopper has been shown to be altered by insecticide application [55]. Enhanced expression of neprilysin and vitellogenin-like genes in GWSS therefore may be related to increased immune response, or alternatively to a reproductive fitness cost, in resistant individuals.

Compared to susceptible GWSS, resistant individuals had a lower expression of cuticle structural and peritrophin-like genes. The cuticle is the first barrier of protection for insects against insecticides. Expression changes in genes predicted to be involved in cuticle structure have been observed in a variety of insect species [16, 27, 28, 56]. While some studies of hemipteran pests have observed an upregulation of cuticle genes, others in *M. persicae* and *Aphis gossypii*, have reported their downregulation such as was seen here for GWSS [24, 27]. While future work is needed to confirm this, one possibility is that these expression changes, whether up or down, are leading to cuticle modifications that could contribute to resistance (e.g. thicker cuticles). Alternatively, the down regulation of these genes may indicate a fitness cost, with resistant individuals prioritizing detoxification mechanisms over barrier protection. Peritrophins are proteins with chitin-binding domains that are an integral part of the peritrophic membrane, which lines the insect gut. The peritrophic membrane is thought to aid in digestion and protection from toxins [57]. Previous work has shown altered expression of peritrophin-like proteins linked to cycloxaprid application [58] and silencing peritrophins can lead to higher imidacloprid susceptibility in termites [59]. While it's unclear here why we observe reduced expression of pertriphonins in resistant individuals, it is possible that these genes have roles in resistance mechanisms through altered gut structure or detoxification. It is also possible that these represent another trade-off related to gut structure and digestion in resistant individuals.

### A lack of broad-scale population structure is consistent with previous work

GWSS has been proliferating in California since its initial invasion in the 1990s, where it was introduced via humans likely through nursery shipments [1, 2]. Given this recent introduction, and the relatively small geographic scale examined here, it is perhaps not surprising to see limited broad-scale population structure, especially when considering coding regions. Previous work by Stenger et al. used a GWSS reovirus, due to its faster rate of evolution, to date the introduction of GWSS to California [2]. Their results pointed at an introduction followed by a bottleneck expansion, which is in agreement with the levels of inbreeding observed here. Our results are also consistent with a study by Smith (2015), who used the mitochondrial cytochrome oxidase I (COI) gene to investigate population structure of GWSS across the United States [60]. Their results showed two distinct groups of haplotypes of GWSS in the United States, a group of populations from east of the Mississippi River including Louisiana, Mississippi, Alabama and Florida, and a group composed of populations west of the Mississippi River from Texas and California. However, they found that neither group of haplotypes had sufficient genetic structuring to further differentiate populations within the two groups. Furthermore, the data supported the hypothesis that GWSS populations in California most likely originated from a source in Texas, arising from random distribution by humans, and not from GWSS from east of the Mississippi River. The subsequent distribution of GWSS throughout California could promote gene-flow within populations that would contribute to the low differentiation and lack of population structure seen here.

## Conclusion

We provide the first, to our knowledge, characterization of the transcriptome of neonicotinoid resistant and susceptible *H. vitripennis*. We identified a suite of candidate genes linked to resistance status including a highly expressed cytochrome P450 (J6590_005969), and observed additional expression patterns supportive of multi-gene roles in resistance mechanisms or fitness trade-offs. While we found no evidence of broad-scale population structure, this may be due to the recent introduction of GWSS to California, relatively small geographic range investigated here, or continued gene flow due to accidental distribution by humans. Follow up work is needed to investigate the specific functional roles and molecular mechanisms responsible for the DEGs identified here, particularly the upregulated cytochrome P450 (J6590_005969), and to confirm whether these DEGs can affect GWSS resistance in vivo. Additionally, future studies should also consider whether obligate and facultative microbial symbionts of GWSS are involved in conferring insecticide resistance (e.g. [61]). We believe that this work serves as a foundation for future studies of insecticide resistance in GWSS and other Hemipteran insects.

## Methods

### Sample collection and sequencing

**Fig. 5:**
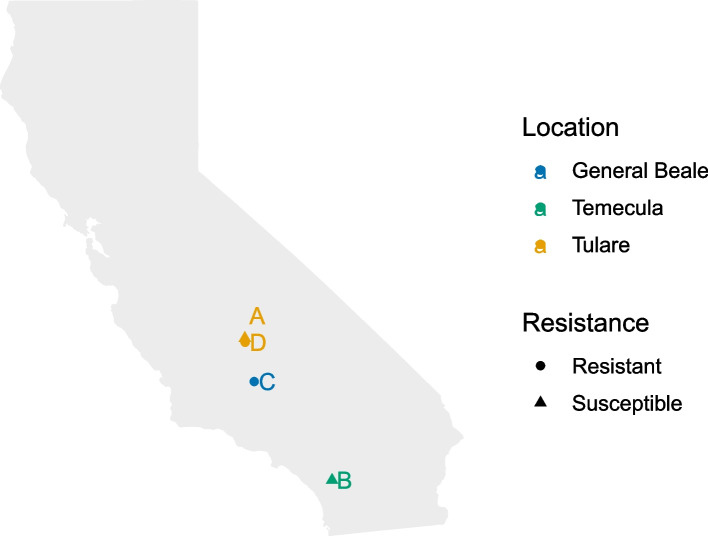
Map depicting collection locations. Map diagram showing California with points depicting different populations. Points are labeled by population (A-D; Table 1), colored by collection location, and shapes are used to display resistance status (circle = resistant, triangle = susceptible)

Sharpshooters were previously collected in August 2019 from California citrus groves in Porterville (Tulare-Organic), Temecula (Temecula-Organic), Bakersfield (General Beale-Organic) and Terra Bella (Tulare-Conventional) (Fig. 5) as part of a multi-year monitoring program and were confirmed to have varying levels of neonicotinoid resistance [9]. Baseline susceptible levels for imidacloprid were determined in 2003 using populations that had never been exposed to imidacloprid. Using a range of imidacloprid doses, insects were treated by topically applying insecticide to their abdomens. Mortality was assessed at 48 h, and LD50s (the lethal dose that results in 50% mortality) derived from the dose–response curves using probit analysis. Resistance was defined when the LD50 of a field population was statistically significant from the LD50 of a susceptible population. However, it was not possible to determine an LD50 for the General Beale-Organic or the Tulare-Conventional populations due to the high levels of resistance. Therefore, a discriminating dose of 500 ng/insect was chosen to distinguish susceptible and resistant insects [9]. In 2020, insects from the General Beale-Organic population were tested at the discriminating dose, resulting in only 16% mortality (FJB unpublished). The latter result continued a consistent pattern of high resistance in GWSS collected from that region of California since the first tests were conducted in 2017. In contrast, tests with insects from the Temecula-Organic population confirmed full susceptibility to imidacloprid in 2019 [9], and in 2020 when the most recent data were determined (FJB unpublished).

Four sharpshooters from each of these four locations were chosen from the 2019 collections to produce a total of 16 sharpshooter transcriptomes (Table 1) [9, 33]. For the resistant locations (Tulare-Conventional and General Beale-Organic), GWSS were treated with imidacloprid to confirm resistance (as described above) and healthy survivors (resistant GWSS) were sequenced. While for the susceptible locations (Tulare-Organic and Temecula-Organic), a proportion of collected GWSS were tested with imidacloprid to confirm susceptibility levels and untreated GWSS from the same collections were sequenced. For each sharpshooter, RNA was extracted from adult prothoracic leg tissue using the Monarch Total RNA Mini Kit (New England Biolabs, Ipswich, MA). Paired-end RNA-Seq libraries were constructed with NEBNext Ultra II Directional RNA prep (New England Biolabs, Ipswich, MA) and sequenced on NovaSeq 6000 to produce an average of 87 M paired reads per library (minimum library 51 M, max library 124 M reads). The RNASeq data is available on NCBI Genbank under BioProject PRJNA717315. Computational scripts associated with analysis in this manuscript are available on GitHub and archived in Zenodo [62].

### Transcriptome expression and functional enrichment

The *H. vitripennis* UCR_GWSS_1.0 assembly and the annotation described previously and available on NCBI at JAGXCG010000000 were used here as a reference [33]. EDTA v.1.9.4 was used to annotate repetitive elements for this assembly using the following parameters: –anno 1 –evaluate 1 –sensitive 1 –step all [63]. Transcriptome reads were aligned against the *H. vitripennis* UCR_GWSS_1.0 reference genome using STAR v.2.7.9a to generate bam files and then these files were summarized at the gene level using featureCounts v.1.6.2 [64, 65]. These count tables were uploaded into R v.4.1.2 and analyzed using the DESeq2 package v.1.34.0 to examine the log_2_ fold change (i.e. differential expression) of genes between insecticide-resistant and susceptible populations [66, 67]. We subsequently focused on genes with Bonferroni corrected *p*-values < 0.05 that had a log_2_ fold change >|2|. Enrichment analysis of Gene Ontology (GO) terms was performed on differentially expressed genes to identify GO terms that were significantly over-enriched (*p* < 0.05). This analysis was performed for each of the three GO classes (i.e., biological processes [BP], molecular functions [MF], and cellular components [CC]). Differential expression and GO enrichment results were visualized in R using clusterProfiler v.4.2.2 [68]. When graphing expression trends, count data was normalized using the variance stabilizing transformation. The R package gggenomes v.0.9.5.9000 was used to visualize genes of interest [69].

Predicted cytochrome P450 genes were aligned using MUSCLE v.3.8.425 [70]. The resulting alignment was then trimmed using the -automated1 option in trimAl v.1.4.1 [71]. A maximum likelihood tree was then built from the trimmed alignment using IQTREE2 v.2.1.3 with 1000 bootstraps [72]. This phylogeny was imported into R for visualization with ggtree v.3.2.1 [73].

### Variant calling

Transcriptome bam files were processed using ‘AddOrReplaceReadGroups’, ‘MarkDuplicates’ and ‘​​SplitNCigarReads’ to assign reads to new sample groups, flag duplicate reads and split reads containing N’s using Picard tools (http://broadinstitute.github.io/picard). The variants (SNVs and indels) were genotyped relative to the H. vitripennis UCR_GWSS_1.0 reference genome using the HaplotypeCaller step in GATK v4.0 [74]. Predicted variants were filtered using GATK’s SelectVariants call with the following parameters: for SNVs, -window-size = 10, -QualByDept < 2.0, -MapQual < 40.0, -QScore < 100, -MapQualityRankSum <  − 12.5, -StrandOddsRatio > 4.0, -FisherStrandBias > 60.0, -ReadPosRankSum <  − 8.0; for indels, -window-size = 10, -QualByDepth < 2.0, -MapQualityRankSum <  − 12.5, -StrandOddsRatio > 10.0, -FisherStrandBias > 200.0, -ReadPosRank <  − 20.0, -InbreedingCoeff <  − 0.8. Variants were subsequently annotated with snpEff [75]. We then used VCFtools v.0.1.16 to investigate missingness across samples using –missing-indv, and further filtered the variant table using –missingness 0.75 and –mac 3 [76]. Resulting variant tables contained 300,365 polymorphic SNVs. Using VCFtools, we converted final VCF files into plink format for some analyses [77].

### Population analysis

Variant tables were imported into R in VCF and plink formats for analysis. We used hierfstat v.0.5–10 to calculate basic population statistics, such as observed and expected heterozygosity, using the basic.stats function [78]. We also used the hierfstat package to calculate the fixation index statistics including F_ST_ and F_IS_ both within and between populations. We then used fastSTRUCTURE to assess broad-scale population structure using K values from 1 to 30 [79]. The ‘chooseK.py’ function was used to assess which K provided the best marginal likelihood value. We also investigated broad-scale structure using a complementary method, LEA, with K values from 1 to 30 across ten iterations [80]. We then performed AMOVA tests on population stratification using poppr v.2.9.3 and ade4 v.1.7–18 [81, 82]. We ran AMOVAs on resistance status (resistant, susceptible) alone and then in nested hierarchies with collection county (Tulare, Temecula, General Beale), population (A-D), or both. Finally, we used OutFLANK v.0.2 to search for and identify F_ST_ outliers that might be linked to resistance status [83].

## Supplementary Information

**Additional file 1: Table s1. **Differentially expressed genes between insecticide-resistant and susceptible GWSS. DESeq2 was used to identify differentially expressed genes (DEGs) between insecticide-resistant and susceptible GWSS populations. We provide here a table of all significant 607 DEGs including their gene ID, whether the gene was upregulated in resistant or susceptible populations, the log_2_ fold change, Bonferonni corrected *p-*value, InterPro annotation, and associated GO terms. DEGs in the table are ordered by *p*-value.

**Additional file 2:** **Figure s1.** Phylogeny of all cytochrome P450s and their relative expression levels. The eight cytochrome P450s that were differentially expressed between insecticide-resistant and susceptible glassy winged sharpshooters, and which had higher expression in resistant individuals, are highlighted in the tree in orange. A heatmap displays the variance stabilized counts for each cytochrome P450 across all sharpshooters sampled.

**Additional file 3: Figure s2. **STRUCTURE results also indicate no broad-scale population structure. STRUCTURE plots for K=1 to K=4 populations. Marginal likelihood is maximized at K = 1.

**Additional file 4: Figure s3. **LEA results support that overall population structure is indicative of weak differentiation. (A) LEA cross-entropy across K=1 to K=30. There is a break in the cross-entropy values at K=16 which is equal to the number of individuals in this study. Cross-entropy was lowest at K = 1. (B) LEA sparse nonnegative matrix factorization predicted ancestry proportions for K=1 to K=4.

## Data Availability

The transcriptome data is publicly available on DDBJ/ENA/GenBank under BioProject PRJNA717315. Computational scripts associated with the analysis of this data are available on GitHub and archived in Zenodo [62].
