# Supplementary figures and images for: Transcriptome and population structure of glassy-winged sharpshooters (Homalodisca vitripennis) with varying insecticide resistance in southern California
Source: BMC Genomics. 2022 Oct 22;23:721. doi: 10.1186/s12864-022-08939-1 (PMC9587601; doi:10.1186/s12864-022-08939-1)

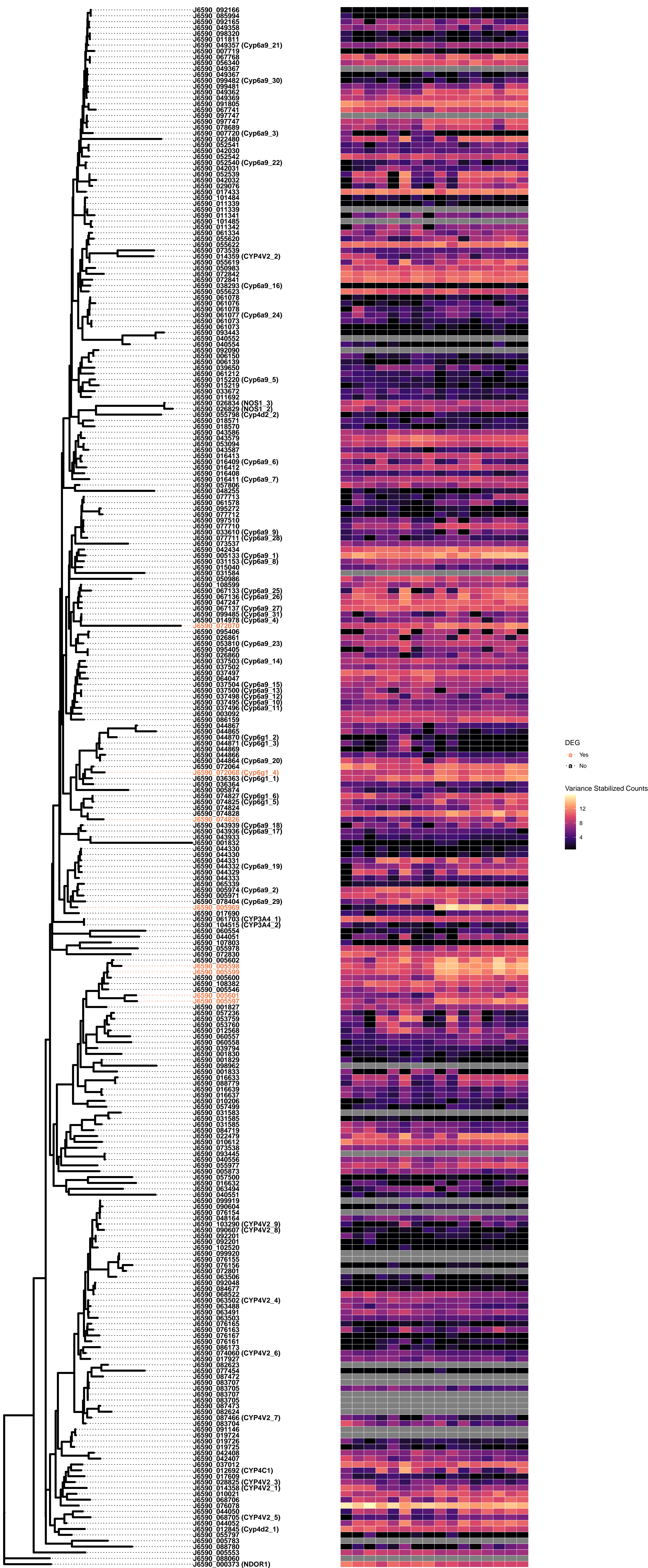

A1 A2 A3 A4 B1 B2 B3 B4 C1 C2 C3 C4 D1 D2 D3 D4

Supplement: Supplementary file 2 — Additional file 2: Figure s1. Phylogeny of all cytochrome P450s and their relative expression levels. The eight cytochrome P450s that were differentially expressed between insecticide-resistant and susceptible glassy winged sharpshooters, and which had higher expression in resistant individuals, are highlighted in the tree in orange. A heatmap displays the variance stabilized counts for each cytochrome P450 across all sharpshooters sampled. [file 12864_2022_8939_MOESM2_ESM.pdf]

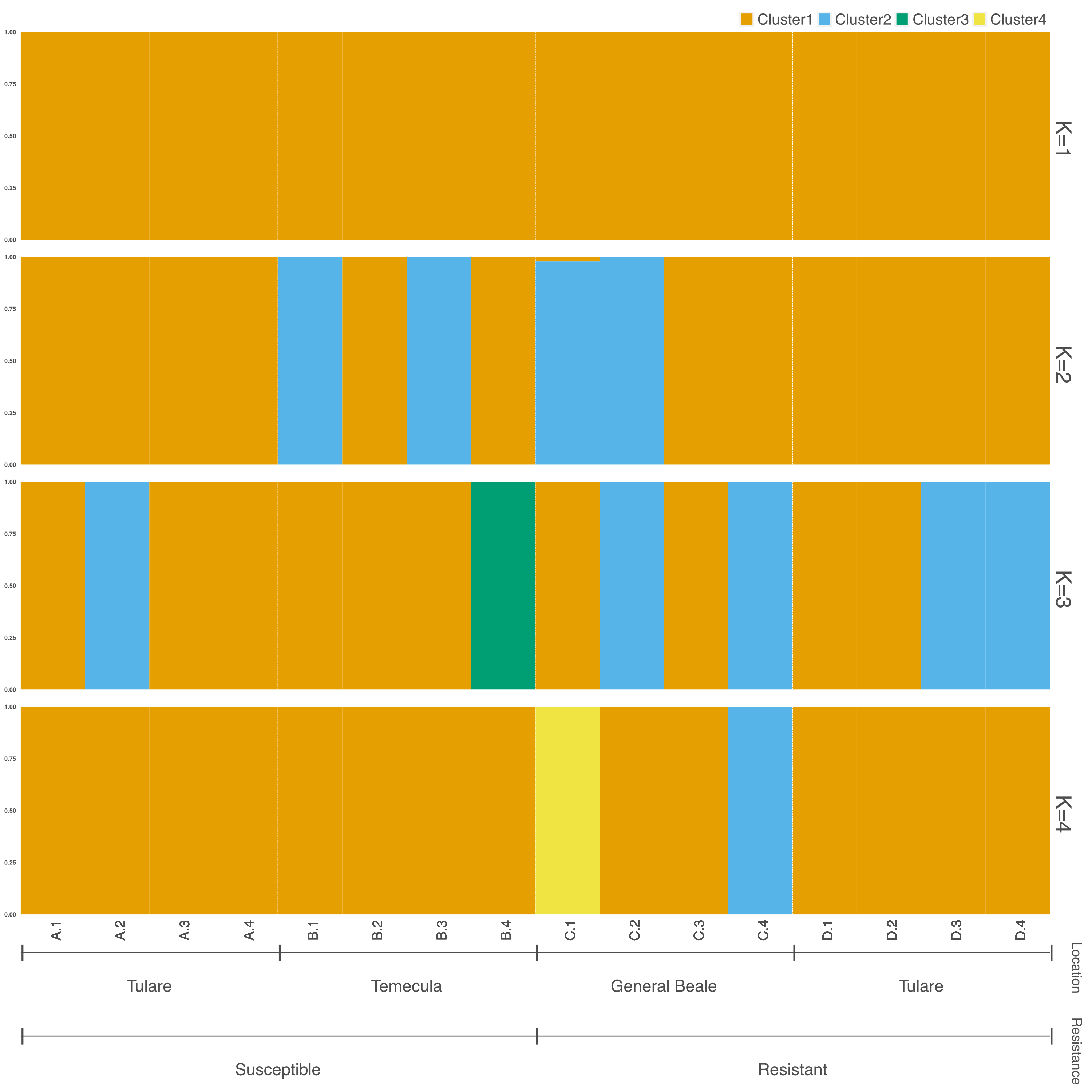

Supplement: Supplementary file 3 — Additional file 3: Figure s2. STRUCTURE results also indicate no broad-scale population structure. STRUCTURE plots for K=1 to K=4 populations. Marginal likelihood is maximized at K = 1. [file 12864_2022_8939_MOESM3_ESM.png]

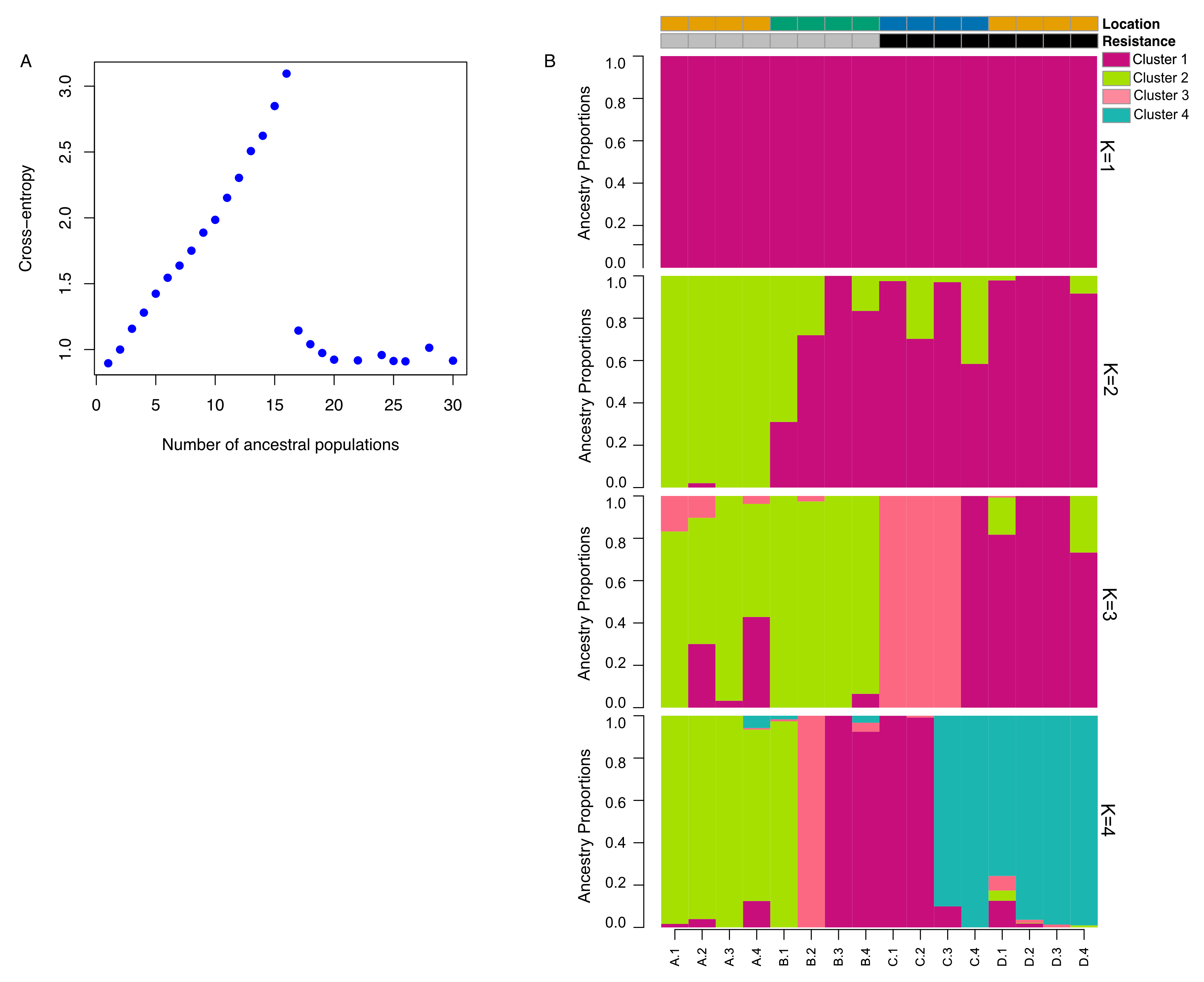

Supplement: Supplementary file 4 — Additional file 4: Figure s3. LEA results support that overall population structure is indicative of weak differentiation. (A) LEA cross-entropy across K=1 to K=30. There is a break in the cross-entropy values at K=16 which is equal to the number of individuals in this study. Cross-entropy was lowest at K = 1. (B) LEA sparse nonnegative matrix factorization predicted ancestry proportions for K=1 to K=4. [file 12864_2022_8939_MOESM4_ESM.png]
